# Nonrigid Medical Image Registration by Finite-Element Deformable Sheet-Curve Models

**DOI:** 10.1155/IJBI/2006/73430

**Published:** 2006-08-13

**Authors:** Jianhua Xuan, Yue Wang, Matthew T. Freedman, Tulay Adali, Peter Shields

**Affiliations:** ^1^ Department of Electrical Engineering and Computer Science, The Catholic University of America, Washington, DC 20064, USA; ^2^ Department of Electrical and Computer Engineering, Virginia Polytechnic Institute and State University, Arlington, VA 22203, USA; ^3^ Department of Oncology and the Lombardi Comprehensive Cancer Center, Georgetown University, Washington, DC 20057, USA; ^4^ Department of Computer Science and Electrical Engineering, University of Maryland, Baltimore County, Baltimore, MD 21250, USA

## Abstract

Image-based change quantitation has been recognized as a promising tool for accurate assessment of tumor's early response to chemoprevention in cancer research. For example, various changes on breast density and vascularity in glandular tissue are the indicators of early response to treatment. Accurate extraction of glandular tissue from pre- and postcontrast magnetic resonance (MR) images requires a nonrigid registration of sequential MR images embedded with local deformations. This paper reports a newly developed registration method that aligns MR breast images using finite-element deformable sheet-curve models. Specifically, deformable curves are constructed to match the boundaries dynamically, while a deformable sheet of thin-plate splines is designed to model complex local deformations. The experimental results on both digital phantoms and real MR breast images using the new method have been compared to point-based thin-plate-spline (TPS) approach, and have demonstrated a significant and robust improvement in both boundary alignment and local deformation recovery.


Hindawi Publishing Corporation
International Journal of Biomedical Imaging
Volume 2006, Article ID 73430, Pages 1–9
DOI 10.1155/IJBI/2006/73430

### Nonrigid Medical Image Registration by Finite-Element Deformable Sheet-Curve Models

**Jianhua Xuan,[1] Yue Wang,[2] Matthew T. Freedman,[3] Tulay Adali,[4] and Peter Shields[3]**

[1] *Department of Electrical Engineering and Computer Science, The Catholic University of America, Washington, DC 20064, USA*
[2] *Department of Electrical and Computer Engineering, Virginia Polytechnic Institute and State University, Arlington, VA 22203, USA*
[3] *Department of Oncology and the Lombardi Comprehensive Cancer Center, Georgetown University, Washington, DC 20057, USA*
[4] *Department of Computer Science and Electrical Engineering, University of Maryland, Baltimore County, Baltimore, MD 21250, USA*

Received 16 August 2005; Revised 5 May 2006; Accepted 18 May 2006

Recommended for Publication by Ming Jiang

Image-based change quantitation has been recognized as a promising tool for accurate assessment of tumor's early response to chemoprevention in cancer research. For example, various changes on breast density and vascularity in glandular tissue are the indicators of early response to treatment. Accurate extraction of glandular tissue from pre- and postcontrast magnetic resonance (MR) images requires a nonrigid registration of sequential MR images embedded with local deformations. This paper reports a newly developed registration method that aligns MR breast images using finite-element deformable sheet-curve models. Specifically, deformable curves are constructed to match the boundaries dynamically, while a deformable sheet of thin-plate splines is designed to model complex local deformations. The experimental results on both digital phantoms and real MR breast images using the new method have been compared to point-based thin-plate-spline (TPS) approach, and have demonstrated a significant and robust improvement in both boundary alignment and local deformation recovery.

Copyright © 2006 Jianhua Xuan et al. This is an open access article distributed under the Creative Commons Attribution License, which permits unrestricted use, distribution, and reproduction in any medium, provided the original work is properly cited.

### 1. INTRODUCTION

Chemoprevention is an established approach to cancer prevention based on 25 years of research [1–3]. The breast cancer prevention trial (BCPT) in the United States recently led the Food and Drug Administration (FDA) to approve tamoxifen as a chemopreventive drug to reduce breast cancer risk [4]. Although the overall benefits of tamoxifen outweigh the overall risks, the risks were found significant in the trials, including increased risks of endometrial cancer, problems related to blood clots, and cataracts [5]. Moreover, many clinical studies have also reported that not all women are protected against breast cancer by tamoxifen. Given the risks and the lack of full protection against breast cancer, some high-risk women are reluctant to use tamoxifen because they want to know whether tamoxifen is effective on their breasts, rather than relying on statistical evidence. Hence, it is clinically important to develop novel approaches to accurately assess early response to tamoxifen.

Previous studies have shown that estrogens increase breast density [6, 7], but the effect on breast density of selective estrogen receptor modulators (SERMs), such as tamoxifen and raloxifene, is unknown. In a previous clinical trial [6], one author of this paper has evaluated the effects of two years of treatment with raloxifene, estrogen, or placebo on breast density using a digitized analysis of mammograms. The main findings of the study can be summarized as the following: (1) the mean breast density was statistically significantly greater in the conjugated estrogens group than it was in the other three groups, and (2) volunteers receiving raloxifene did not increase breast density after two years of treatment. In the study, breast density, as measured by mammography, is determined by the relative amounts of fat and fibroglandular tissue present. As discussed in [6], since estrogen action works through estrogen receptors that are present in glandular tissue, breast density measured on glandular tissue would more directly indicate the response to SERM chemoprevention. However, mammography dose not allow us to accurately measure the density of glandular tissue in practice.

In this study, we use gadolinium-enhanced MR imaging method to differentiate glandular tissue from fibroglandular tissue, and measure the breast density on the glandular tissue. In the light of the antiestrogenic properties of tamoxifen, that is, reduction of circulating growth factors and inhibition of angiogenesis [8], we propose to quantitatively measure changes of breast density and breast vascular density in glandular tissue to assess early response to tamoxifen. With such quantitative measurements, we can determine whether the drug is effective for a patient, early and individually. To compare the glandular tissue volumes between pre- and postcontrast MR images, nonrigid registration should be performed to align two MR images and recover local deformations.

Many nonrigid registration methods have been proposed in recent years [9–11], and can be categorized into intensity-based or feature-based approaches [12–15]. When using gadolinium-enhanced MR images, feature-based approaches offer advantages over intensity-based registration methods since geometric features—points, curves, and surfaces—are consistent between pre- and postcontrast MR images while intensity values are not. By establishing feature correspondences, deformations can be recovered by nonlinear interpolation methods such as the thin-plate-spline (TPS) method [13].

Registration accuracy often depends on the availability of a large number of features. Point-based registration methods typically use anatomical landmark points as the features to register images [13]. Although anatomical landmarks are the most robust features, the number of landmarks is usually limited in MR breast images. Moreover, if local deformations are confined in certain regions, the landmarks have to be well placed over those regions. It is therefore reasonable to use the boundaries of anatomical objects as features to recover local deformations [14, 16].

Several approaches have been proposed that used boundaries or curves as matching features [14, 16–18]. Moshfeghi proposed an elastic matching method for registration of multimodality images [14]. The method is based on Burr's elastic contour matching algorithm and a Gaussian smoothed deformation model that estimates the deformations over the whole image [19, 20]. To reduce the computational complexity, Davatzikos et al. proposed a two-step approach to recover deformations based on boundary mapping, that is, a homothetic mapping of boundaries followed by an elastic deformation transformation (EDT) [16].

However, elastic matching method often suffers from the following problems: (1) since no physical model (or constraint) is imposed on the curve deformation, the resulted curve matching is considered only an ad hoc solution; and (2) the Gaussian smoothed deformation model is an over-simplified model when facing the complex local deformations existed in MR breast images. For example, although Davatzikos' method is a very efficient algorithm resulting in a great reduction in computational complexity, the key assumption behind is homothetic mapping, that is, a mapping by a uniform scaling of its length and arbitrary length-preserving bending [16]. Such an assumption cannot hold in MR breast images in that the participated deformations are relatively large, therefore homothetic mapping could produce large curve matching error.

In this paper, we propose deformable sheet-curve models to overcome the problems associated with the existing methods. Deformable curves are used to obtain a reliable matching of the curves using physically constrained deformations, and a deformable sheet with the energy functional of thin-plate splines is used to model complex local deformations between the images.

### 2. THEORY AND METHOD

In image registration a nonlinear transformation $\mathbf{U}(\mathbf{x}) = (U^x(x, y), U^y(x, y))$ is used to map the first image to the second image. We refer to the function $\mathbf{U}(\mathbf{x})$ as the deformation, which can be modeled as either thin-plate splines [13] or elastic sheets [16]. Under the assumption that we have $K$ pairs of corresponding boundaries (or curves), $(C_{1i}, C_{2i})$, $i = 0, \ldots, K-1$, we use deformable curves to model the deformation of curves in the two images (denoted as $\mathbf{U}_c$), and a deformable sheet to model the deformation between two images (denoted as $\mathbf{U}_s$). We propose a deformable sheet-curve model shown in Figure 1 to recover the deformation $\mathbf{U}_s$ through matching the deformable curves and deforming the deformable sheet iteratively.

A deformable sheet-curve model can be presented in variational and finite-element forms. We denote the deformation of the sheet by $\mathbf{U}_s(x, y, t) = (U_s^x(x, y, t), U_s^y(x, y, t))$, where $(x, y)$ is the bivariate material coordinate and $t$ is the time index. Similarly, the deformation of the curve can be denoted by $\mathbf{U}_c(s, t) = (U_s^x(s, t), U_s^y(s, t))$, where $s$ is the univariate material coordinate of the curve. The strain energy $E$ can be found to characterize the deformable material of the sheet or the curve as an instance of the spline function. At the center of our method, the continuum mechanical equation [21]

$$\mu \frac{\partial^2 \mathbf{U}}{\partial t^2} + \gamma \frac{\partial \mathbf{U}}{\partial t} + \frac{\delta E(\mathbf{U})}{\delta \mathbf{U}} = \mathbf{f}(\mathbf{U}) \quad (1)$$

governs the nonrigid motion of the sheet (curve) in response to the extrinsic force $\mathbf{f}(\mathbf{U})$, where $\mu$ is the mass density function of the deformable sheet (curve) and $\gamma$ is the viscosity function of the ambient medium. The third term on the left-hand side of the equation is the variational derivative of the strain energy functional $E$, the internal elastic force of the sheet (curve).

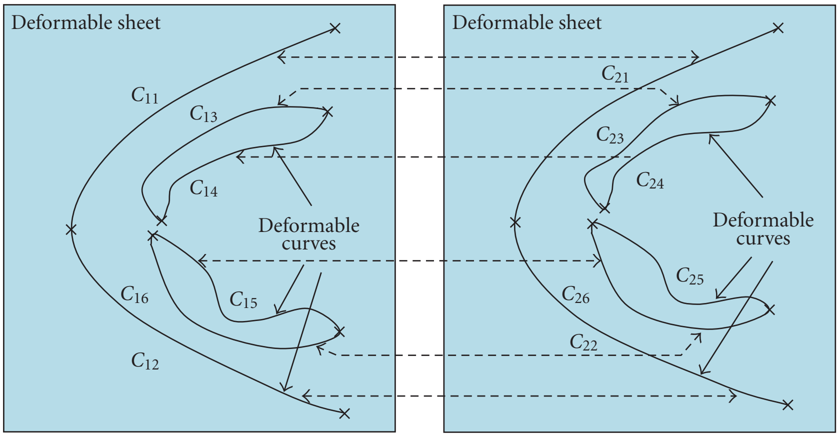

Figure 1: Configuration of the deformable sheet-curve model.

### 2.1. Variational modeling

The deformable energy for the deformation $\mathbf{U}_s(x, y, t)$ of the sheet is defined by

$$E_s(x,y) = \iint \left( w_{10}\left|\frac{\partial \mathbf{U}_s}{\partial x}\right|^2 + 2w_{11}\left|\frac{\partial \mathbf{U}_s}{\partial x}\right|\left|\frac{\partial \mathbf{U}_s}{\partial y}\right| + w_{01}\left|\frac{\partial \mathbf{U}_s}{\partial y}\right|^2 + w_{20}\left|\frac{\partial^2 \mathbf{U}_s}{\partial x^2}\right|^2 + 2w_{22}\left|\frac{\partial^2 \mathbf{U}_s}{\partial x \partial y}\right|^2 + w_{02}\left|\frac{\partial^2 \mathbf{U}_s}{\partial y^2}\right|^2 \right) dx\,dy, \quad (2)$$

where the weights $w_{01}$, $w_{11}$, and $w_{10}$ control the tensions of the sheet (stretching energy), and $w_{02}$, $w_{22}$, and $w_{20}$ control its "rigidities" by weighting the bending energy (see [22] for a detailed discussion on maximal rigidity). Accordingly, the deformable energy for the deformation $\mathbf{U}_c(s, t)$ of the curve is given by

$$E_c(x,y) = \iint \left( w_1\left|\frac{\partial \mathbf{U}_c}{\partial s}\right|^2 + w_2\left|\frac{\partial^2 \mathbf{U}_c}{\partial s^2}\right|^2 \right) ds. \quad (3)$$

The weight $w_1$ controls the tension along the curve (stretching energy), and $w_2$ controls its rigidity (bending energy).

The dynamics of the deformable sheet-curve model is defined by the following Lagrangian motion equations:

$$\mu\frac{\partial^2 \mathbf{U}_c^i}{\partial t^2} + \gamma\frac{\partial \mathbf{U}_c^i}{\partial t} + \frac{\delta E(\mathbf{U}_c^i)}{\delta \mathbf{U}_c^i} = \mathbf{f}_c^i, \quad i = 0,\dots,K-1,$$

$$\mu\frac{\partial^2 \mathbf{U}_s}{\partial t^2} + \gamma\frac{\partial \mathbf{U}_s}{\partial t} + \frac{\delta E(\mathbf{U}_s)}{\delta \mathbf{U}_s} = \mathbf{f}_s, \quad (4)$$

where $\mathbf{f}_s$ and $\mathbf{f}_c^i$ are the external forces applied on the sheet and the curve, respectively. The registration process is an iterative procedure by repeating the following two steps.

(1) *Boundary deformation recovery by deformable curve matching*: deformable curves use the corresponding curves in the second image to define the external forces ($\mathbf{f}_c^i$, $i = 0,\dots,K-1$). The deformations of the curves ($\mathbf{U}_c^i(s,t)$, $i = 0,\dots,K-1$) can then be obtained by solving Lagrangian motion equations of the curves.

(2) *Image deformation recovery by deforming the deformable sheet*: the deformable sheet uses the new curve positions ($C_{1i}^{\text{new}} = C_{1i} + \mathbf{U}_c^i$, $i = 0,\dots,K-1$) to define the external forces ($\mathbf{f}_s$). The local deformation of the image can be obtained by solving Lagrangian motion equation of the sheet.

The final deformation $\mathbf{U}_s$ is obtained when the energies of the deformable sheet-curve model reach their minima.

We define the external forces $\mathbf{f}_c^i$ to reflect the mismatch between the two corresponding curves $(C_{1i}, C_{2i}) : \mathbf{f}_c^i = D(C_{1i}, C_{2i})$, where $D(C_{1i}, C_{2i})$ is the Gaussian weighted Euclidean distance of each point on the first curve to the nearest point on the second curve. After we recover the curve deformations $\mathbf{U}_c^i$, we obtain the new curve positions as $C_{1i}^{\text{new}} = C_{1i} + \mathbf{U}_c^i$, $i = 0,\dots,K-1$. Then we use Gaussian weighted Euclidean distance to define the external forces for the deformable sheet, that is, $\mathbf{f}_s = D((C_{1i}, C_{1i}^{\text{new}}), i = 0,\dots,K-1)$.

### 2.2. Finite element modeling

We use a finite-element method to compute the numerical solutions of $\mathbf{U}_s$ and $\mathbf{U}_c$. We first tessellate the continuous material domain, $(x, y)$ for the sheet and $s$ for the curve, into a mesh of $m$ element subdomains $D_j$. We then approximate $\mathbf{U}$ by a weighted sum of continuous basis functions $\mathbf{N}_i$ (so-called shape functions): $\mathbf{U} \approx \mathbf{U}^h = \sum_{i=1}^{n} \mathbf{U}_i\mathbf{N}_i = \mathbf{Na}$, where $\mathbf{U}_i$ is a vector of nodal variables associated with mesh node $i$, $\mathbf{N}_i$ is a vector of shape functions associated with node $i$, $n$ is the number of nodes of an element, $\mathbf{a}^T = [\mathbf{U}_1^T,\dots,\mathbf{U}_n^T]$, and $\mathbf{N} = [\mathbf{N}_1,\dots,\mathbf{N}_n]$. The shape functions $\mathbf{N}_i$ are fixed in advance and the nodal variables $\mathbf{U}_i$ are the unknowns. The motion equation (4) can then be discretized as

$$\mathbf{M}\frac{\partial^2 \mathbf{a}}{\partial t^2} + \mathbf{C}\frac{\partial \mathbf{a}}{\partial t} + \mathbf{K}\mathbf{a} = \mathbf{F}, \tag{5}$$

where $\mathbf{M}$ is the mass matrix, $\mathbf{C}$ is the damping matrix, $\mathbf{K}$ is the stiffness matrix, and $\mathbf{F}$ is the forcing matrix. $\mathbf{M}$, $\mathbf{C}$, and $\mathbf{F}$ can be obtained as follows:

$$\begin{aligned} \mathbf{M} &= \iint \mu \mathbf{N}^T \mathbf{N}\, dx\, dy, \\ \mathbf{C} &= \iint \gamma \mathbf{N}^T \mathbf{N}\, dx\, dy, \\ \mathbf{F} &= \iint \mathbf{N}^T \mathbf{f}\, dx\, dy. \end{aligned} \tag{6}$$

To compute $\mathbf{K}$, we have the following equation:

$$\mathbf{K} = \iint \left(\mathbf{N}_s^T \boldsymbol{\alpha} \mathbf{N}_s + \mathbf{N}_b^T \boldsymbol{\beta} \mathbf{N}_b\right) dx\, dy, \tag{7}$$

where

$$\begin{gathered} \mathbf{N}_s = \left[\frac{\partial \mathbf{N}}{\partial x}, \frac{\partial \mathbf{N}}{\partial y}\right]^T, \qquad \mathbf{N}_b = \left[\frac{\partial^2 \mathbf{N}}{\partial x^2}, \frac{\partial^2 \mathbf{N}}{\partial x \partial y}, \frac{\mathbf{N}}{\partial y^2}\right]^T, \\ \boldsymbol{\alpha} = \begin{bmatrix} w_{10} & w_{11} \\ w_{11} & w_{01} \end{bmatrix}, \qquad \boldsymbol{\beta} = \begin{bmatrix} w_{20} & 0 & 0 \\ 0 & w_{22} & 0 \\ 0 & 0 & w_{02} \end{bmatrix}. \end{gathered} \tag{8}$$

### 2.2.1. Deformable sheet element

The deformable sheet consists of a set of connected triangular elements. Barycentric coordinates are the natural choice for defining shape functions over a triangular domain, since a unifying representation of different triangles can be achieved [23]. Barycentric coordinates $(L_1, L_2, L_3)$ are defined by the following mapping with material coordinate $(x, y)$:

$$\begin{bmatrix} x \\ y \\ 1 \end{bmatrix} = \begin{bmatrix} x_1 & x_2 & x_3 \\ y_1 & y_2 & y_3 \\ 1 & 1 & 1 \end{bmatrix} \begin{bmatrix} L_1 \\ L_2 \\ L_3 \end{bmatrix}, \tag{9}$$

where $(x_1, y_1)$, $(x_2, y_2)$, and $(x_3, y_3)$ are the coordinates of three vertex locations of a triangle.

We construct a 9-degree-of-freedom (DOF) triangular element with its position and first parametric partial derivatives at each triangle vertex. The shape functions for the 9-DOF triangle are [24]

$$\mathbf{N}_1^{9^T} = \begin{bmatrix} L_1 + L_1^2 L_2 + L_1^2 L_3 - L_1 L_2^2 - L_1 L_3^2 \\ c_3\left(L_1^2 L_2 + 0.5 L_1 L_2 L_3\right) - c_2\left(L_1^2 L_3 + 0.5 L_1 L_2 L_3\right) \\ -b_3\left(L_1^2 L_2 + 0.5 L_1 L_2 L_3\right) + b_2\left(L_1^2 L_3 + 0.5 L_1 L_2 L_3\right) \end{bmatrix}. \tag{10}$$

The triangle's symmetry in barycentric coordinates can be used to generate the shape function for the second and third nodes in terms of the first. To generate $\mathbf{N}_2^9$ use the above equations but add a 1 to each index so that 1→2, 2→3, and 3→1. The $\mathbf{N}_3^9$ functions are generated by adding another 1 to each index. By defining 9 DOFs of the triangular element as $\mathbf{a}^T = [\mathbf{U}_1, \mathbf{U}_{1x}, \mathbf{U}_{1y}, \mathbf{U}_2, \mathbf{U}_{2x}, \mathbf{U}_{2y}, \mathbf{U}_3, \mathbf{U}_{3x}, \mathbf{U}_{3y}]$, we can approximate the $\mathbf{U}_s$ as

$$\mathbf{U}_s^h = \mathbf{N}^9 \mathbf{a} = \begin{bmatrix} \mathbf{N}_1^9 & \mathbf{N}_2^9 & \mathbf{N}_3^9 \end{bmatrix} \mathbf{a}. \tag{11}$$

### 2.2.2. Deformable curve element

The finite element of the curve has 4 DOFs for the two nodes located atthe ends of the curve segment. The DOFs at each node are the position and tangent of the node. The deformation of the curve segment can be approximated as the weighted sum of a set of Hermite polynomials [23]:

$$\mathbf{U}_c \approx \mathbf{U}_c^h(s) = \sum_{i=0}^{3} \mathbf{U}_c^i \mathbf{N}_i, \tag{12}$$

where $\mathbf{U}_c^i$, $i = 0, \ldots, 3$, are nodal variables and $\mathbf{N}_i$, $i = 0, \ldots, 3$, are given as follows:

$$\begin{aligned} N_0 &= 1 - 3\left(\frac{s}{h}\right)^2 + 2\left(\frac{s}{h}\right)^3, \\ N_1 &= h\left(\frac{s}{h} - 2\left(\frac{s}{h}\right)^2 + \left(\frac{s}{h}\right)^3\right), \\ N_2 &= 3\left(\frac{s}{h}\right)^2 - 2\left(\frac{s}{h}\right)^3, \\ N_3 &= h\left(-\left(\frac{s}{h}\right)^2 + \left(\frac{s}{h}\right)^3\right), \end{aligned} \tag{13}$$

where $h$ is the parametric element length.

### 2.2.3. Numerical integration

The deformable sheet-curve model can be stabilized during the matching process if its motion is critically damped to minimize vibrations. Critical damping can be achieved by appropriately balancing the mass and damping distributions. A simple way of eliminating vibration while preserving useful dynamics is to set the mass density in (1) to zero, thus reducing (5) to

$$\mathbf{C}\frac{\partial \mathbf{a}}{\partial t} + \mathbf{K}\mathbf{a} = \mathbf{F}. \tag{14}$$

This first-order dynamic system governs the model that has no inertia and comes to rest as soon as all the forces balance. We integrate (14) using an explicit first-order Euler method [24]. The method begins with a simple forward difference approximation. By considering extrapolation from time with the forward difference approximation

$$\frac{\partial \mathbf{a}}{\partial t} = \frac{\mathbf{a}(t + \Delta t) - a(t)}{\Delta t}, \tag{15}$$

(14) then becomes

$$\mathbf{C}\mathbf{a}(t + \Delta t) = (\mathbf{C} - \Delta t \mathbf{K})\mathbf{a}(t) + \Delta t \mathbf{F}. \tag{16}$$

Thus we obtain the updating formula as follows:

$$\mathbf{a}(t+\Delta t) = (\mathbf{I} - \Delta t\mathbf{C}^{-1}\mathbf{K})\mathbf{a}(t) + \Delta t\mathbf{C}^{-1}\mathbf{F}. \qquad (17)$$

It is well known that finite difference methods for initial-value systems yield expressions very similar to the above results obtained by finite-element schemes [25]. A noteworthy distinction is that the coefficient matrix for finite differencing is diagonal in the usual difference approximation. This leads, in the forward difference approximation, to an efficient algorithm for solving the problem. In the finite-element method, due to the sparseness of $\mathbf{C}$, it is difficult to compute $\mathbf{C}^{-1}$ since complicated singular decomposition methods have to be used. Fortunately, a physical solution exists in computational mechanics, namely, the "lumping" procedure, to overcome such difficulties. The idea can be physically interpreted as replacing the continuous material with the distributed mass, that is, concentrated material with "lumped" mass ("beads") at the nodes. In practice, there are several ways to perform such a "lumping" procedure—for example, using modified shape functions or different numerical integral methods [24]. Among those, the easiest way is to keep only the diagonal coefficients and is adopted here in solving the motion equations of the deformable sheet-curve model.

### 3. EXPERIMENTAL RESULTS

Deformable sheet-curve models were implemented using a finite-element method using 9-DOF triangular sheet elements and 4-DOF curve elements. The deformable curves are used to match boundaries, and the deformable sheet is modeled as a thin-plate spline to recover the local deformation. The registration process is an iterative procedure by repeating the following two steps: (1) the deformations of the curves ($\mathbf{U}_c^i$) are obtained by solving Lagrangian motion equations of the curves with the external forces ($\mathbf{f}_c^i$) defined by the corresponding curves, and (2) the deformation of the sheet ($\mathbf{U}_s$) is obtained by solving Lagrangian motion equation of the deformable sheet with the external forces ($\mathbf{f}_s$) defined by the new curve positions (derived from $\mathbf{U}_c^i$). The final deformation of the whole image is recovered when the energies of the deformable sheet-curve model reach their minima. In our experiments, we applied the deformable sheet-curve models to both simulated data and real breast MR images to demonstrate the performance of the method in recovering boundary deformation and image deformation.

### 3.1. *Simulation result*

Digital phantoms are generated and used to validate the proposed boundary-based registration approach in a controlled manner with known truth. As shown in Figure 2, the boundaries in the first image are depicted in solid line while those in the second image are in dashed line. In our approach, we use deformable curves to model the boundaries and match them by recovering the deformation iteratively. The recovered boundary deformation demonstrates an excellent matching between the initial and target boundaries with a mean square error (MSE) less than 2%, where the parameters (i.e., the weights in (3)) are experimentally chosen to obtain a good overall matching performance. The deformable sheet works cooperatively with deformable curves to infer the image deformation iteratively. As we can see from Figure 2, the recovered image deformation is constrained by the thin-plate-spline functional. The digital phantom study therefore provides compelling evidence that deformable sheet-curve models can achieve accurate boundary matching and complex image deformation modeling.

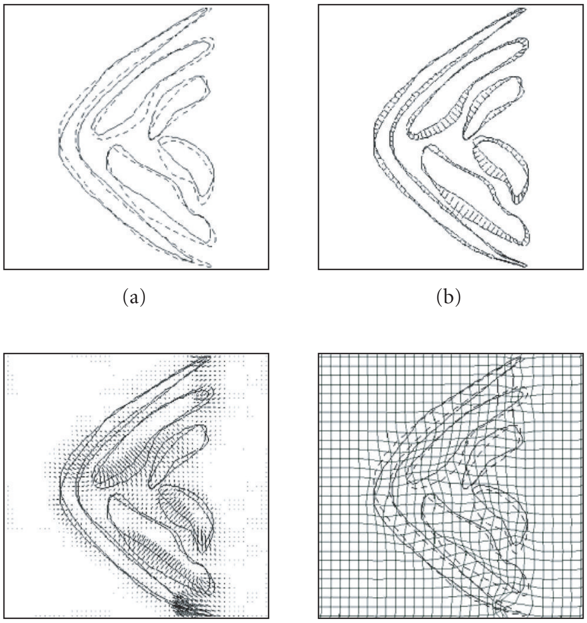

(a) (b) (c) (d)

Figure 2: A simulation result deformation recovered by the deformable sheet-curve model: (a) curve features, (b) recovered boundary deformation, (c) recovered image deformation, and (d) recovered image deformation in mesh representation.

### 3.2. *Registration of breast MR images*

We have further applied our deformable sheet-curve model to register real contrast-enhanced sequential MR breast images to assess early response to chemoprevention. The data set of this study consists of serial studies in 29 women at high risk of breast cancer who received either tamoxifen or no drug. MR images are acquired with contrast agent (gadolinium) at 0, 3, and 6 months along with biopsies for histopathology, RNA, and gene analysis at Lombardi Cancer Center (LCC) of the Georgetown University Medical Center. As of October of 2004, we have completed baseline testing on 26 enrolled women, three MR scans on 26 women and biopsies on 22 women.

MR images are acquired using Siemens' Magnetom Vision with magnetic field strength of 1.5 Tesla. One 3D MR scan of the patient results in 64 images with pixel resolution at $512 \times 512$, where pixel spacing is 0.36 mm in both X- and Y-directions, and slice thickness is 2.0 mm in Z-direction. Some example images are shown in Figure 3, where pre- and

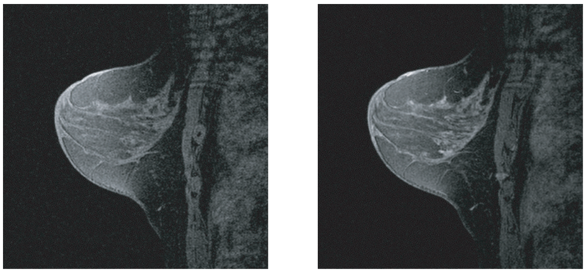

(a)

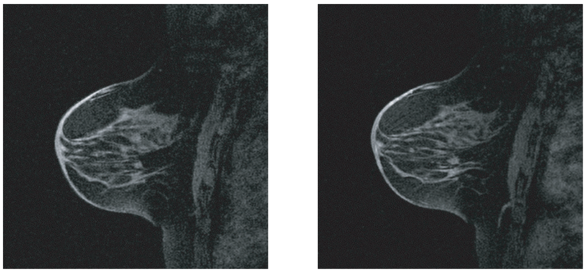

(b)

Figure 3: Sample MR pre- and postcontrast images at (a) 0 month and (b) 3 months: (left) precontrast image and (right) postcontrast image.

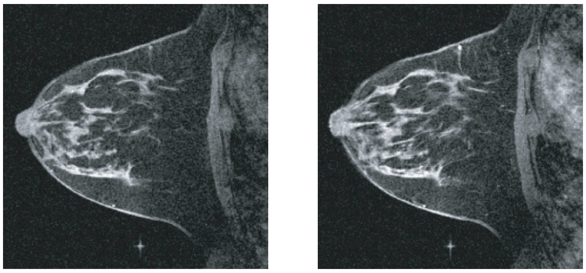

(a) (b)

Figure 4: A pair of MR breast images: (a) precontrast image, and (b) postcontrast image.

postcontrast images at 0 and 3 months are shown in Figures 3(a) and 3(b), respectively.

We use fibroglandular tissue as the reference object to register pre- and postcontrast MR breast images, see Figure 4 for an example. The regions of fibroglandular tissue are extracted using a stochastic model-based segmentation method based on the standard finite normal model (SFNM) and a contextual Bayesian relaxation labeling (CBRL) procedure [3]. The segmentation result is shown in Figure 5 with all the regions of fibroglandular tissue identified in both pre- and postcontrast images. Then all the corner points on the boundaries of fibroglandular tissue are extracted (see Figure 5). By establishing the corner point correspondence, we can partition the boundaries of fibroglandular tissue into corresponding curves in pre- and postcontrast images. These

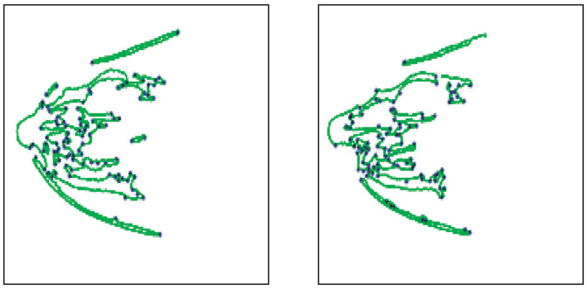

(a) (b)

Figure 5: Extracted fibroglandular tissue with corner pointed detected: (a) precontrast image, and (b) postcontrast image.

curves serve as the features in the registration of pre- and postcontrast MR breast images.

The deformable curves with 4-DOF finite elements are used to model the extracted boundaries of fibroglandular tissue, and the deformable sheet with 9-DOF finite elements is then used to model image deformation. The registration process is a cooperative procedure by repeating boundary matching and image deformation inferring. The deformable curve matching gives us the boundary deformation, and the recovered boundary deformation is then used to deform the deformable sheet to derive image deformation. In our finite-element implementation, we use numerical integration method described in Section 2 to solve the Lagrangian motion equations. The final deformation of the image is recovered when the energies of the deformable sheet-curve model reach their minima. Figure 6 shows the extracted regions of glandular tissue by subtracting the registered precontrast image from the postcontrast image.

As a comparison, we show the extracted regions of glandular tissue using the registration result of the point-based TPS method in Figure 7. As we can see, many false regions of glandular tissue are resulted from the misalignment of the boundaries of fibroglandular tissue due to the limited number of the available feature points (25 feature points in this experiment). The boundary misalignment can be clearly seen in Figure 7(b), where two regions of fibroglandular tissue are registered by the point-based TPS method. Figure 7(a) shows the boundary registration result using our deformable sheet-curve model, which demonstrates a great improvement in boundary alignment compared with that of the point-based TPS method. The experimental results show that our deformable sheet-curve model can provide an expected improvement in registration accuracy regarding both boundary alignment and local deformation recovery.

### 4. CONCLUSION

We have developed deformable sheet-curve models for nonrigid medical image registration. The registration method supported by our deformable sheet-curve models is a feature-based approach in that (1) deformable curves are used to model curve features—the boundaries of objects,

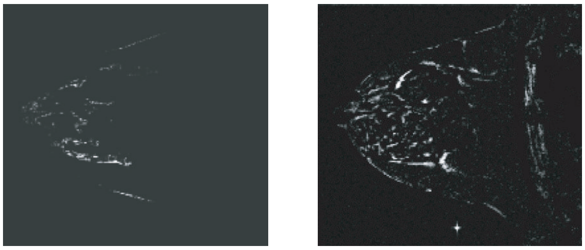

(a) (b)

Figure 6: Extracted glandular tissue: (a) using the deformable sheet-curve model, and (b) using TPS with feature points alone.

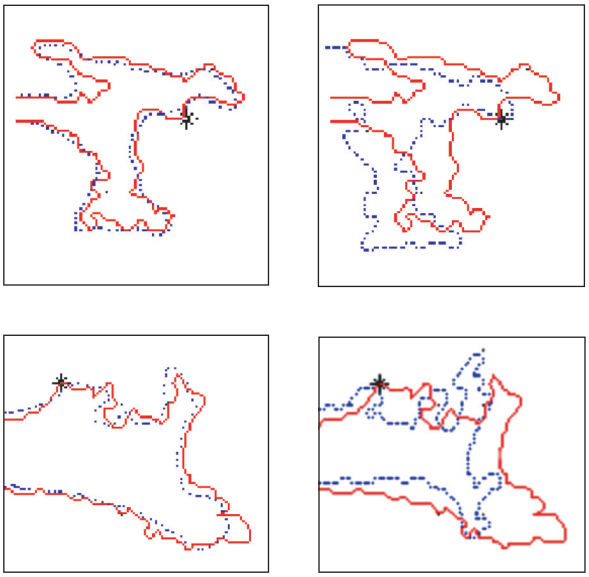

(a) (b)

Figure 7: Registered boundaries of two selected regions: (a) using the deformable sheet-curve model, and (b) using TPS with feature points alone.

and (2) the thin-plate-spline sheet is used to model the local deformation over the image. The deformable sheet-curve model is also a dynamic system that is governed by Lagrangian motion equations. The model has been implemented in finite-element method with 9-DOF triangular sheet elements and 4-DOF curve elements. By solving Lagrangian motion equations iteratively, we can recover the deformation over the whole image. We have applied the deformable sheet-curve model to register MR breast images for the assessment of early response to chemoprevention. The experimental results have demonstrated a significant improvement in the registration accuracy with reliable boundary alignment and accurate local deformation recovery. We believe that our comparative studies provide useful information on the utility of the proposed method for nonrigid image registration. Given the difficulty of the task, while the optimality of the method may be data or modality dependent, we would expect it to be an important tool in change detection across temporal image sequences.

### ACKNOWLEDGMENT

This work was supported in part by the Department of Defense under Grant DAMD17-03-0448.

### REFERENCES

[1] G. J. Kelloff, "Perspectives on cancer chemoprevention research and drug development," *Advances in Cancer Research*, vol. 78, pp. 199–334, 1999.

[2] S. M. Lippman, J. J. Lee, and A. L. Sabichi, "Cancer chemoprevention: progress and promise," *Journal of the National Cancer Institute*, vol. 90, no. 20, pp. 1514–1528, 1998.

[3] D. S. Alberts, O. M. Colvin, A. H. Conney, et al., "Prevention of cancer in the next millennium: report of the chemoprevention working group to the American Association for Cancer Research," *Cancer Research*, vol. 59, no. 19, pp. 4743–4758, 1999.

[4] B. Fisher, J. P. Costantino, D. L. Wicherham, et al., "Tamoxifen for prevention of breast cancer: report of the national surgical adjuvant breast and bowl project P-1 study," *Journal of the National Cancer Institute*, vol. 90, no. 18, pp. 1371–1388, 1998.

[5] K. Meister, "Chemoprevention of breast cancer," American Council on Science and Health, March 2000, http://www.acsh.org.

[6] M. T. Freedman, J. S. Martin, J. O'Gorman, et al., "Digitized mammography: a clinical trial of postmenopausal women randomly assigned to receive raloxifene, estrogen, or placebo," *Journal of the National Cancer Institute*, vol. 93, no. 1, pp. 51–56, 2001.

[7] G. A. Greendale, B. A. Reboussin, A. Sie, et al., "Effects of estrogen and estrogen-progestin on mammographic parenchymal density. Postmenopausal Estrogen/Progestin Interventions (PEPI) Investigators," *Annals of Internal Medicine*, vol. 130, pp. 262–269, 1999.

[8] U. Veronesi, P. Maisonneuve, A. Costa, et al., "Prevention of breast cancer with tamoxifen: preliminary findings from the Italian randomise trial among hysterectomised women," *Lancet*, vol. 352, pp. 93–101, 1998.

[9] L. G. Brown, "A survey of image registration techniques," *ACM Computing Surveys*, vol. 24, no. 4, pp. 325–376, 1992.

[10] J. S. Duncan and N. Ayache, "Medical image analysis: progress over two decades and the challenges ahead," *IEEE Transactions on Pattern Analysis and Machine Intelligence*, vol. 22, no. 1, pp. 85–106, 2000.

[11] P. A. van den Elsen, E. J. D. Pol, and M. A. Viergever, "Medical image matching - a review with classification," *IEEE Engineering in Medicine and Biology Magazine*, vol. 12, no. 1, pp. 26–39, 1993.

[12] R. Bajcsy and S. Kovačič, "Multiresolution elastic matching," *Computer Vision, Graphics, and Image Processing*, vol. 46, no. 1, pp. 1–21, 1989.

[13] F. L. Bookstein, "Principal warps: thin-plate splines and the decomposition of deformations," *IEEE Transactions on Pattern Analysis and Machine Intelligence*, vol. 11, no. 6, pp. 567–585, 1989.

[14] M. Moshfeghi, "Elastic matching of multimodality medical images," *CVGIP: Graphical Models and Image Processing*, vol. 53, no. 3, pp. 271–282, 1991.

[15] Y. Wang, K. Woods, and M. McClain, "Information-theoretic matching of two point sets," *IEEE Transactions on Image Processing*, vol. 11, no. 8, pp. 868–872, 2002.

[16] C. Davatzikos, J. L. Prince, and R. N. Bryan, "Image registration based on boundary mapping," *IEEE Transactions on Medical Imaging*, vol. 15, no. 1, pp. 112–115, 1996.

[17] M. Droske and M. Rumpf, "A variational approach to nonrigid morphological registration," *SIAM Journal on Applied Mathematics*, vol. 64, no. 2, pp. 668–687, 2004.

[18] M. Droske and W. Ring, "A Mumford-Shah level-set approach for geometric image registration," Preprint Series DFB-SPP 1114, preprint 99, April 2005, http://www.math.uni-bremen.de/zetem/DFG-Schwerpunkt/preprints/pdf/099.pdf.

[19] D. J. Burr, "Elastic matching of line drawings," *IEEE Transactions on Pattern Analysis and Machine Intelligence*, vol. 3, no. 6, pp. 708–713, 1981.

[20] D. J. Burr, "A dynamic model for image registration," *Computer Graphics & Image Processing*, vol. 15, no. 2, pp. 102–112, 1981.

[21] D. S. Chandrasekharaiah and L. Debnath, *Continuum Mechanics*, Academic Press, New York, NY, USA, 1994.

[22] S. L. Keeling and W. Ring, "Medical image registration and interpolation by optical flow with maximal rigidity," *Journal of Mathematical Imaging and Vision*, vol. 23, no. 1, pp. 47–65, 2005.

[23] G. Celniker and D. Gossard, "Deformable curve and surface finite-elements for free-form shape design," *Computer Graphics*, vol. 25, no. 4, pp. 257–266, 1991.

[24] O. C. Zienkiewicz, *The Finite Element Method*, McGraw-Hill, New York, NY, USA, 3rd edition, 1967.

[25] D. M. Young, *Iterative Solutions of Large Linear System*, Academic Press, New York, NY, USA, 1971.

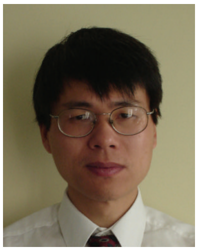

**Jianhua Xuan** received his Ph.D. degree in electrical engineering and computer science from the University of Maryland in 1997. He received his B.S., M.S., and Ph.D. degrees from University of Zhejiang, China, in 1985, 1988, and 1991, respectively, all in electrical engineering. Currently, he is an Assistant Professor in the Department of Electrical Engineering and Computer Science at The Catholic University of America. His research interests include biomedical image analysis, cellular and molecular imaging, computational bioinformatics, systems biology, intelligent information systems, visual intelligence, computer vision, information visualization, and machine learning. He is a recipient of several NIH/DoD/NASA grant awards, and leads a groups effort in cancer research and biomedical research. He is also a Member of the Editorial Board for BioMedical Engineering OnLine.

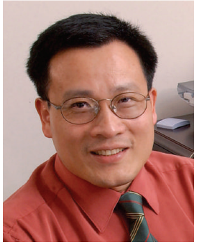

**Yue Wang** received his B.S. and M.S. degrees in electrical and computer engineering from Shanghai Jiao Tong University in 1984 and 1987, respectively. He received his Ph.D. degree in electrical engineering from University of Maryland Graduate School in 1995. In 1996, he was a Postdoctoral Fellow at Georgetown University School of Medicine. From 1996 to 2003, he was an Assistant and later Associate Professor of electrical engineering at The Catholic University of America, Washington, DC. Since 2003, he has been an Associate Professor of electrical, computer, and biomedical engineering at Virginia Polytechnic Institute and State University, Arlington, Va. He is also an affiliated faculty member of radiology and radiological science with the Johns Hopkins University. He became a Fellow of The American Institute for Medical and Biological Engineering (AIMBE) in 2004. His research interests focus on intelligent computing, machine learning, pattern recognition, statistical visualization, and advanced imaging and image analysis, with applications to molecular analysis of human diseases.

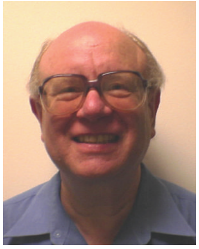

**Matthew T. Freedman** is a Radiologist and an Associate Professor of oncology, Lombardi Comprehensive Cancer Center, Georgetown University Medical Center, Washington, DC. He is Director of the Advanced Cancer Imaging Division of the ISIS Imaging Science Research Center at Georgetown. He is, at Georgetown, the lead Radiologist for two national cancer institute studies of the effectiveness of screening for lung cancer. He does research in the imaging of rodent mammary glands studying the effect of dietary changes on mammary gland development and early carcinogenesis. He studies problems in the detection of lung cancer and the effect of computer aided detection (CAD) of lung cancer on radiologist performance. He provides medical guidance to graduate students in the biomedical engineering/computer science programs at The Catholic University of America and the Advanced Research Institute of Virginia Tech.

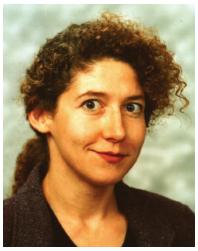

**Tulay Adali** received the Ph.D. degree in electrical engineering from North Carolina State University, Raleigh, in 1992 and joined the faculty at the University of Maryland, Baltimore County (UMBC), Baltimore, the same year. She is currently a Professor in the Department of Computer Science and Electrical Engineering at UMBC. She worked in the organization of a number of international conferences and workshops including the IEEE International Conference on Acoustics, Speech, and Signal Processing (ICASSP), the IEEE International Workshop on Neural Networks for Signal Processing (NNSP), and the IEEE International Workshops on Machine Learning for Signal Processing (MLSP). She was the general cochair for the NNSP workshops 2001–2003 and the technical chair of the MLSP workshops 2004–2006. She is the past chair and current Member of the MLSP Technical Committee, and is serving on the IEEE publications board and the IEEE Signal Processing Society conference board. Her research interests are in the areas of statistical signal processing, machine learning for signal processing, biomedical data analysis (functional MRI, MRI, PET, CR, ECG, and EEG), bioinformatics, and signal processing for optical communications. She is the recipient of a 1997 National Science Foundation CAREER Award.

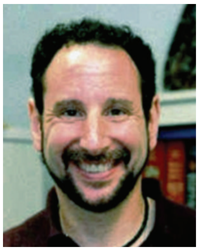

**Peter Shields** is currently a Full Professor in the Departments of Oncology and Medicine at Georgetown University. He is the Associate Director for Cancer Control and Population Sciences in the Lombardi Comprehensive Cancer Center, and the Program Leader for Cancer Genetics and Epidemiology. Also, he is the Director of the Division of Cancer Genetics and Epidemiology in the Department of Oncology. Dr. Shields' focus of research is on gene-environment interactions for cancer risk.

The laboratory component of his research develops new biomarkers of cancer risk, while his epidemiological component accrues special populations. A major initiative that is led by Dr. Shields is a Breast Cancer Center of Excellence. This program is a multi-investigator and multidisciplinary approach to understand why alcohol drinking causes breast cancer. Dr. Shields has over 120 peer-reviewed publications, included in high profile journals. He serves, or has served, on editorial boards of major journals such as Carcinogenesis and Cancer, Epidemiology, Biomarkers and Prevention. At Georgetown University, he is a Member of the Lombardi Comprehensive Cancer Center Executive Committee, the oversight committee for the Tumor Biology program, and the M.D.-Ph.D. program.

